# A Review and Perspective of Techniques for Autonomous Robotic Ultrasound Acquisitions

**DOI:** 10.3390/s26072081

**Published:** 2026-03-27

**Authors:** Yanding Qin, Lele Dang, Fan Ren, Zhuomao Li, Lijun Duan, Hongpeng Wang, Jianda Han

**Affiliations:** 1 College of Artificial Intelligence, Nankai University, Tianjin 300350, China; qinyd@nankai.edu.cn (Y.Q.); 2120230541@mail.nankai.edu.cn (L.D.); renfan@mail.nankai.edu.cn (F.R.); 1120250310@mail.nankai.edu.cn (Z.L.); 2 Shenzhen Research Institute of Nankai University, Shenzhen 518083, China; 3 Department of Endocrinology and Metabolism, Tianjin First Center Hospital, Tianjin 300190, China

**Keywords:** medical robotics, ultrasound imaging, robotic ultrasound acquisition, autonomous robotic system, medical image processing

## Abstract

Ultrasound (US) imaging is a widely used diagnostic method in clinics. Real-time-generated US images are used for rapid diagnosis without harm to patients. The quality of US imaging highly depends on the skill of the physician due to the differences among physicians. Techniques for autonomous robotic ultrasound (AU-RUS) acquisitions are expected to become an effective means to improve the level of US diagnosis, reduce the workload of physicians, and improve the standardization of US imaging quality. This paper aims to summarize the current research status of techniques for AU-RUS acquisitions, and to discuss the research trends and challenges regarding related technologies. Firstly, the techniques for AU-RUS acquisitions and systems are outlined. The techniques for teleoperated or autonomous US acquisitions are briefly discussed. Representative RUS acquisition systems are introduced. Then, the current research status of AU-RUS acquisitions is reviewed from four research directions: force sensitivity and control, scanning path-planning and positioning, US treatment guidance, and US image processing technology and quality assessment optimization. This review provides a decision-oriented autonomy perspective by mapping typical methods to workflow components across the stages of perception, decision-making, and execution. We identify major deployment bottlenecks, including safety-verifiable autonomy and failure recovery, motion compensation under deformation, and the lack of standardized, clinically meaningful US image quality metrics. Finally, the shortcomings of current research are summarized and analyzed, and the research trends and challenges for AU-RUS acquisitions are prospected.

## 1. Introduction

B-mode ultrasound (US) imaging began to be used for medical diagnosis in the 1940s [1]. Its principle is based on transmitting US waves into the human body via a US transducer while simultaneously receiving the reflected echo signals. Since the echoes carry information about the biological tissues, intuitive imaging of the corresponding tissues can be achieved by analyzing the distribution and intensity of the echoes, allowing physicians to assess the health of the tissues for diagnostic purposes [2]. Compared to other medical imaging modalities, such as computed tomography (CT) and magnetic resonance imaging (MRI), US imaging offers the distinct advantages of portability, real-time capability, safety, and low cost [3,4]. As a result, it is widely applied in various clinical fields, such as obstetrics and gynecology, cardiology, urology, and gastroenterology [5,6,7,8,9,10,11].

Despite its unique advantages, US imaging also has a notable weakness: the examination must be performed when the US probe is in “close” contact with the patient’s body. Furthermore, image quality is highly dependent on the contact status [12], placing high demands on the physician’s skill and experience. During US examination, the physician must first identify the correct scanning area, and then continuously move the US probe while maintaining adequate contact pressure and constantly adjusting its position to optimize image quality. Additionally, the physician must use their other hand to continuously adjust the parameters of the US machine. This diagnostic process relies entirely on the physician’s skill in integrating anatomical prior knowledge with the US images to assess imaging quality and form a diagnosis. Such a heavily operator-dependent procedure has become a bottleneck, limiting the broader application and adoption of US imaging.

The need for manual operation makes it difficult to adapt to intraoperative navigation applications, and even harder to integrate them with other intelligent surgical tools. Additionally, US image quality highly depends on the physician’s skill and experience. Reliable diagnostic outcomes are often determined by the proficiency of the specialized physician. The current lack of quantitative evaluation standards for US imaging quality can lead to discrepancies in diagnosis for the same case among different physicians [13]. Furthermore, the repetitive, mechanical motions involved in the daily examination of a large number of cases impose significant mental and physical strain on physicians. The most common issues are permanent fatigue-related injuries to local muscles and skeletal structures [14,15], which can also contribute to imaging defects caused by human operation. Moreover, it is also worth noting that during infectious disease outbreaks, the challenge of reducing infection risks associated with contact-based examinations is a prominent issue for conventional manual US procedures [16,17]. Finally, the lengthy and costly training cycle for physicians, coupled with the uneven distribution of medical resources, results in a shortage of highly skilled US physicians in remote and impoverished regions. This situation also occurs in various extreme environments, e.g., disaster zones, scientific expeditions in harsh conditions, and space missions, where deploying a sufficient number of specialized US physicians is exceptionally difficult.

With the rapid advancement of robotics and artificial intelligence (AI), medical robots have gradually demonstrated flexibility and human–robot friendliness distinct from traditional industrial robots. Progress in soft materials and sensor technology has endowed medical robots with a level of dexterity and perceptual capabilities comparable to that of the human arm. Extensive clinical studies have confirmed that medical robots have already shown significant advantages in clinical diagnosis, surgical procedures, postoperative rehabilitation, and home care [18,19,20]. Therefore, integrating robotic technology with US image acquisition holds the potential to overcome many challenges associated with manual US imaging. This approach is expected to realize standardized and consistent US images and has emerged as a prominent research direction in the field of medical-engineering integration. This paper will review relevant research work from three perspectives: robotic ultrasound (RUS) acquisition systems, core enabling technologies, challenges and prospects.

Different from existing surveys focusing on system summaries, this review emphasizes autonomy as decision-making capability across the full US acquisition workflow. We clarify which workflow components are system-led, shared, or human-led at different autonomy levels. We further distinguish engineering autonomy from clinically acceptable diagnostic autonomy and discuss clinical readiness and translation barriers. We summarize the cross-cutting bottlenecks and prioritize near-term barriers to deployment.

## 2. Overview of RUS Acquisition Systems

The RUS acquisition system aims to replace the physician, holding the US probe with a robotic arm to obtain medical diagnostic-quality US images while ensuring patient safety. In recent years, with the rapid development of collaborative robotic arms, force-sensing and control technologies, and image processing techniques, various RUS systems have been developed. Based on the level of involvement of the US physician, these systems can be categorized into three types: remote-controlled RUS (RC-RUS), semi-autonomous RUS (SA-RUS), and autonomous RUS (AU-RUS). Some representative RUS systems are listed in Table 1.

To move beyond an involvement-based description, we interpret autonomy in a decision-oriented way by identifying who closes the loop for key workflow components. A typical RUS workflow can be decomposed into target localization and initial pose selection, force regulation and acoustic coupling maintenance, image quality assessment and acceptance, scan completion and recovery from failures, and physician supervision. From this perspective, RC-RUS usually relies on the physician for most perception and decision steps, while SA-RUS automates specific components, such as force regulation or partial positioning, while keeping the physician in the loop for acceptance and supervision. AU-RUS aims to close more loops, including localization, quality-aware adjustments, and completion decisions under safety constraints. We further distinguish engineering-level autonomy from clinically acceptable diagnostic autonomy. Engineering autonomy mainly refers to the safe and robust execution of scanning primitives, such as stable contact, force-limited motion, and trajectory tracking. Clinically acceptable diagnostic autonomy additionally requires quality-aware decision-making with clinically aligned acceptance criteria, uncertainty monitoring, validated failure detection and recovery behaviors, and evaluation endpoints such as repeatability and diagnostic agreement. Therefore, a system can be mechanically autonomous without being diagnostically autonomous, which is particularly important for interpreting autonomous scanning results in clinical workflows.

RC-RUS represents a significant research direction. Such systems typically consist of an expert console, a robotic arm to operate the US probe, and a software controller that maps the expert’s motions to the robotic arm. In a decision-oriented view, the physician primarily closes the loop for target localization, scan-planning, and image acceptance, while the robot mainly provides motion reproduction and basic safety/force-limiting functions. In 2009, Nakadate et al. [21] developed the first 2D RUS acquisition system, WTA-1RII, for carotid blood flow measurement. The core components include a US probe, a custom-designed six-degrees-of-freedom (DOF) parallel robotic arm, a passive robotic arm, and a joystick-based master controller. As an initial attempt, this system adopts a teleoperation mode and incorporates a passive structure to provide a safety margin. Since it relies on the operator at the master console to ensure both safety and US image quality, the requirements for autonomous robotic control are relatively low.

**Figure 1:**
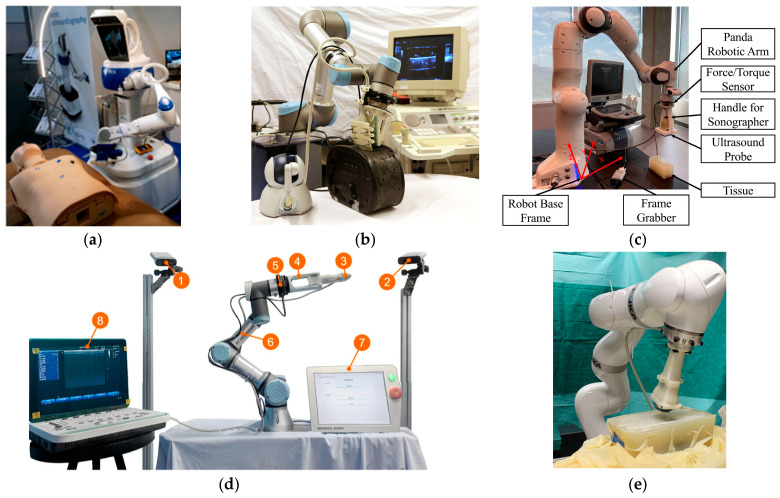
Representative robotic systems for US acquisitions. (**a**) ReMeDi system used in a cardiac exam [22]; (**b**) teleoperated US system with haptic device [29]; (**c**) robot-assisted system for automatic tissue scanning [33]; (**d**) AU-RUS system for thyroid scanning [34]; (**e**) 3D RUS system [38].

As shown in Figure 1a, the ReMeDi robot [22] allows the physician to perform US diagnostics using a set of input devices, including a haptic interface for manipulating the robotic arm and a dedicated keyboard for operating the US machine. The system features a user-friendly human–robot interface and has successfully enabled remote echocardiography examinations. Commercially available system solutions that are currently in use include the MGI system [23] and the MELODY system [24]. The MGI system features high positioning accuracy, high flexibility, safety force protection, and real-time US imaging. Notably, Wang et al. [25] utilized the MGI system to perform RC-RUS examinations on COVID-19 patients, thereby reducing the risk of infection for healthcare providers. The MELODY system features three active DOFs and three passive DOFs, requiring a physician to be on-site for coarse positioning. It has been successfully applied in the US examinations of over 300 patients, covering areas such as cardiac, abdominal, and obstetric imaging [26,27,28]. As shown in Figure 1b, Mathiassen at al. [29] presented an RUS based on a commercial UR5 manipulator. This system integrates robotic motion control with US imaging, demonstrating the feasibility of using a collaborative robot for assisted or automated scanning tasks. Siao et al. [30] developed a system integrating a force sensor, a LiDAR device on the robotic arm, and an adhesive mechanism. This system transforms the robot’s coordinate system into a smartphone coordinate system, enabling physicians to remotely control the robotic arm via their smartphone. A built-in force sensor monitors whether the applied force exceeds safe limits during the procedure, ensuring patient safety and ultimately facilitating RC-RUS examinations.

SA-RUS further incorporates human–robot collaboration and semi-autonomous strategies. It retains the physician’s role in high-level decision-making (e.g., defining the clinical target and acceptance criteria) while delegating specific workflow components such as contact force and coupling maintenance, fine pose adjustment, and safety constraint enforcement to robotic assistance. Therefore, SA-RUS can be mechanically autonomous in its execution while still being physician-led in terms of quality acceptance and diagnostic responsibility. For instance, Mustafa et al. [31] utilized a commercial robotic arm for US acquisition, shifting the research focus towards enhancing the autonomy of the acquisition process. This system established a preliminary overall architecture for an “autonomous” RUS system, i.e., robotic arm + US probe + force sensor + optical sensor. The system utilized an image recognition algorithm to achieve abdominal recognition, thereby determining the starting position and the area requiring scanning for liver US imaging, and preliminarily realized autonomous scanning. Although this work cannot guarantee that the acquired US images meet diagnostic requirements, it validated the feasibility of AU-RUS acquisition. Subsequently, Ma et al. [32] developed a pulmonary RUS system consisting of a 7-DOF robotic arm, a US probe, and an RGB-D camera. This system estimated patient posture and identified the target acquisition area based on DensePose, achieving AU-RUS acquisitions. However, the image quality still required optimization through manual adjustment of the probe’s pose. Huang et al. [13] developed an SA-RUS system for the carotid artery, consisting of a 7-DOF robotic arm, an RGB-D camera, a US probe, and a control console. The system divided the neck scanning procedure into pre-scanning and intraoperative scanning phases. The initial pose and trajectory for the pre-scanning phase were determined based on RGB-D images, while the intraoperative scanning employed hybrid force–position control for implementation. This enabled the system to perform transverse/longitudinal scans of the neck. However, the initial pose and the path selection for the pre-scanning phase still required assistance from a specialized physician. As shown in Figure 1c, Akbari et al. [33] developed an RUS system featuring real-time image-based force adjustment, with the aim of enabling safer US examinations during the COVID-19 pandemic. By combining automatic robotic scanning with online US image quality assessment and force regulation, their method enhanced physical distancing while maintaining scanning performance.

AU-RUS aims to achieve autonomous US acquisition by enabling the robot to plan and execute scanning motions without continuous human teleoperation. More importantly, higher autonomy also implies closing additional loops, such as target localization, quality-aware pose adaptation, scan completion decisions, and failure detection and recovery under safety constraints. However, autonomy in motion-planning and execution does not necessarily imply clinically acceptable diagnostic autonomy, which further requires validated quality metrics and clinically aligned endpoints. As shown in Figure 1d, Su et al. [34] proposed an AU-RUS system for thyroid examination, consisting of a 6-DOF robotic arm equipped with a linear array US probe, a 6-DOF force/torque sensor, and an RGB-D camera. This utilizes human skeleton keypoint recognition for initial positioning and combines reinforcement learning with force feedback to accomplish thyroid target searches. It also employs Bayesian optimization to adjust the US probe pose online, thereby enhancing imaging quality and scanning completeness. Experiments on human subjects demonstrated its potential to achieve image quality and nodule information extraction comparable to that of manual examination.

To enhance image quality, Zielke et al. [35] developed an RUS system for thyroid volume measurement consisting of a 7-DOF robotic arm and a US probe. Based on the acquired US images, a neural network is employed for image segmentation. According to the segmentation results, a scanning trajectory was defined to guide the US probe’s movement, which improved the repeatability and consistency of thyroid lobe volume measurements. However, due to the sensitive anatomical location of the thyroid gland, the initial position of the probe in this system still required manual determination by a physician. Shah et al. [36] developed an RUS system integrated with a 6-DOF robotic arm and a 2D US probe. This system focused on 3D reconstruction technology of the carpal arch based on conventional 2D US imaging, enabling 3D morphological assessment of the carpal arch. The approach of this system holds referential significance for the reconstruction and analysis of other anatomical features in the human body using 2D US images. Tan et al. [37] proposed a system consisting of a US probe, a dual robotic-arm system, a multi-structured light system, a human–robot interaction system, and a flexible US probe clamping device. They designed an end-to-end scanning strategy along with a closed-loop force control strategy to achieve repeatability in breast US imaging. Zielke et al. [35] integrated online segmentation and image appearance feedback into a robotic scanning closed-loop for the thyroid volume measurement. This system comprises a 6-DOF robotic arm and a US probe. The robot guides its motion based on real-time segmentation results to accomplish stable acquisition, thereby significantly reducing measurement errors introduced by physician variability. However, its application objective leans more towards standardized volume estimation than a comprehensive diagnostic procedure.

**Table 1:** Summary of robotics systems for US acquisition.

	Year	Robotic Arm	US Device	Additional Sensor	Technical Features	Application
[13]	2024	Franka Panda (7-DOF)	Running care Smart 3. Linear probe.	RGB-D camera;	Two-phase workflow; Image servoing for path updating; Compliance control to ensure contact safety.	Carotid artery
[23]	2009	Parallel robot (6-DOF)	Pro Sound II (ALOKA); Linear probe.	/	Structural design for probe positioning.	Carotid artery
[30]	2024	Not provided	Linear probe.	Force sensor.	Smartphone-based remote control with ROS/communication architecture; Combined closed-loop pressure-sensing for safety monitoring.	Not specified
[31]	2013	Mitsubishi MELFA RV-1 (6-DOF)	Hitachi.	Camera; Force sensor.	Upper abdominal region recognition and liver localization via image processing algorithms.	Liver
[32]	2021	Franka Panda (7-DOF)	Linear probe.	RGB-D camera.	Scanning region localization using DensePose; Probe 3D pose estimation via RGB-D camera; Real-time constant force control with Franka Panda.	Lung
[36]	2021	Denso (6-DOF)	Acuson S2000 (Siemens); Linear probe.	/	Region of Interest extraction via US image feature detection; 3D reconstruction of the carpal arch combined with point cloud data.	Carpal arch
[37]	2022	Franka Panda (7-DOF)	Linear probe.	RGB-D camera; Force sensor.	3D point cloud-based autonomous breast identification and path-planning with pose–force hybrid control.	Breast
[38]	2017	KUKA LBR iiwa (7-DOF)	Ultrasonix RP (Analogic); Convex probe.	RGB-D camera.	Trajectory-planning based on CT images and acoustic window optimization.	Heart
[39]	2018	Lechuang mechanism (3-DOF)	Ultrasonix RP (Analogic); Convex probe.	RGB-D camera; Force sensor.	3D contour acquisition and translation path-planning from RGB-D images; 3D reconstruction via cubic Bézier curve interpolation.	Not specified
[40]	2018	Epson C4-A601S (6-DOF)	Ultrasonix RP (Analogic); Linear probe.	RGB-D camera; Force sensor.	3D contour acquisition from RGB-D images; Probe pose determination via surface normal alignment; Bézier curve interpolation for 3D reconstruction.	Not specified
[41]	2025	Not provided	Philips EPIQ 7G; X6-1 probe.	RGB-D camera; Force sensor.	Low-cost force-sensing and control to enable stable acoustic coupling and consistent scanning.	Not specified
[42]	2016	KUKA LBR iiwa (7-DOF)	Ultrasonix RP (Analogic); Convex probe.	RGB-D camera.	MRI to body surface registration via coherent point drift algorithm; Image quality estimation and online force estimation via confidence maps.	Abdominal aortic aneurysm
[43]	2025	Franka Panda (7-DoF)	SonoHealth D5CL wireless probe.	RGB-D camera; Force sensor.	Visual servoing navigation combined with force control enables automatic scanning while enhancing consistency/repeatability.	Not specified
[44]	2023	Not provided	2D US probe.	Force sensor.	Integrated force/torque measurement and force control for safe, stable automated scanning.	Not specified
[45]	2022	KUKA LBR Med7 (7-DOF)	2D US probe.	RGB-D camera; Force sensor.	Full-coverage scanning path-planning and stable interactive control; Continuous and stable autonomous scanning via pose–force hybrid control.	Breast
[46]	2025	Not provided	Not Provided.	RGB-D camera.	Point cloud-guided path-planning for kidney scanning.	Kidney
[47]	2025	Franka Panda (7-DOF)	Telemed MicrUs EXT-1H.	RGB-D camera.	RA-UNet-based path-planning for automatic 3D scanning.	Musculoskeletal
[48]	2024	Diana 7 Med (7-DOF)	Philips Affiniti 30; Linear probe.	RGB-D camera; Force sensor.	Autonomous scanning based on visual servoing navigation; Combination of local image quality assessment with recovery control.	Carotid artery

Compared with 2D US imaging, 3D US imaging enables the 3D visualization of target structures. Any slice obtained from 3D US can be reviewed by physicians, providing more precise 3D morphobiological measurements [49] and offering more accurate references for diagnosis [50]. However, 3D US is more sensitive to the applied force and tissue deformation [51,52], leading to poor repeatability and poor standardization for physician-held 3D US probes. In contrast, robotic 3D US imaging fulfils an urgent need and shows broad medical application prospects, making it a key research focus.

The system developed by Göbl et al. [38] consists of a 7-DOF robotic arm, an RGB-D camera, and a US probe, as shown in Figure 1e. The RGB-D camera is positioned above the patient to register CT images to the world coordinate system. The scanning path is automatically planned in preoperative CT data to cover the region of interest inside the patient, with optimization of the acoustic window based on estimations of internal anatomical structures and acoustic transmission. This system used acoustic window quality as the evaluation criterion, resulting in higher coverage of the anatomical target area compared to simple planning approaches.

To address the poor repeatability in physician-performed US acquisition, Kojcev et al. [53] compared the consistency of thyroid lobe length measurements obtained from 3D US images acquired automatically by a robotic system versus those from 2D US scans performed by a physician. Their system employed a 7-DOF robotic arm with an RGB-D camera mounted at its end-effector. Using the point cloud data of the body surface acquired by the RGB-D camera, a scanning trajectory for the thyroid lobe was defined. Compliant behavior was achieved through impedance control, followed by 3D volume reconstruction, with measurements subsequently performed by a physician. Compared to physician-operated US, the RUS system yielded more repeatable and highly consistent measurement results. Jiang et al. [54] addressed the significant tissue deformation caused by probe pressure in 3D US imaging via a patient-specific stiffness-based method. The system consists of a 7-DOF robotic arm with a torque sensor and a linear US probe attached to its flange. It records the contact force and probe pose during palpation to estimate the stiffness of nonlinear tissues. US images are used to compute pixel displacement, characterizing tissue deformation under varying forces. Ultimately, the acquired US images are calibrated to correct deformation and achieve repeatable image acquisition. Regarding challenges in US examination, such as large variations in 3D fetal pose and poor image quality, Chen et al. [55] proposed a novel 3D fetal pose estimation framework, which effectively improves the accuracy of fetal US localization and pose estimation.

US examination protocols vary significantly across different organs. Consequently, 2D or 3D RUS systems are often tailored to specific, personalized US examination applications, lacking universal evaluation metrics. Hence, we will proceed from a technical research perspective, establishing corresponding evaluations based on a summary of the technical framework to assess and characterize existing RUS systems.

## 3. Current Developments on AU-RUS Acquisitions

This section will review the current research status of RUS acquisition from a technical perspective. This includes force sensitivity and control, scanning path-planning and positioning, US treatment guidance, and US image processing technology and quality assessment optimization, as detailed in Table 2. Patient safety is the paramount prerequisite during AU-RUS acquisition. Under the condition of ensured safety, balancing patient comfort with imaging quality places higher demands on the precision and sensitivity of robotic force control. The degree of automation in the acquisition process is closely related to the robot’s localization of the scanning area and the planning technology of the scanning path. Furthermore, the effectiveness evaluation of AU-RUS acquisition technology is strongly correlated with the assessment and optimization of imaging quality and the structural accuracy evaluation of 3D reconstruction.

### 3.1. Force Sensitivity and Control

Based on clinical experience from physician-performed US acquisition, it is understood that continuous contact between the US probe and the patient must be maintained. An appropriate force must be applied to obtain clear images while ensuring patient safety. Chatelain et al. [79,80,86] investigated the relationship between US image confidence values and contact force magnitude. Their research results indicate that inadequate contact forces affect acoustic coupling, resulting in insufficient image confidence and poor image quality. As contact force increases, the confidence value stabilizes after reaching a certain threshold. This suggests that beyond this point, the correlation between contact force magnitude and image quality weakens, while too high a contact force may cause discomfort or even harm to the patient. Zhang et al. proposed a path smoothing optimization method based on the force–strain regression of the breast tissue deformation. Further, they introduced online updating of the contact force by monitoring US image confidence, aiming to maintain good coupling and stable imaging under a lower desired force [87]. Based on the above analyses, precise force-sensing and control constitute a significant research direction in US acquisition systems.

**Figure 2:**
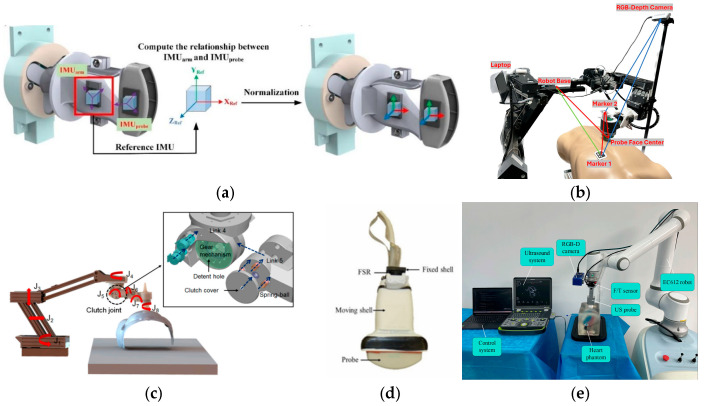
Representative force control methods in RUS acquisition systems: (**a**) dual-side IMU sensor structure [88]; (**b**) a cost-effective US force sensor and force control system [41]; (**c**) force-limiting clutch joint [63]; (**d**) instrumented probe-force measurement [64]; (**e**) RUS scanning system with integral adaptive admittance control [98].

Some researchers have integrated force sensor with the flange of the robot or position it on the US probe to detect the contact force in real time. Huang et al. [39,40] positioned two force sensors on the sides of the probe. The probe’s pose is automatically adjusted to maintain the contact force within the range of 1~8 N. To address the angular misalignment between the robotic arm and the probe caused by the US probe vibration, as shown in Figure 2a, Wang et al. [88] employed a dual-side IMU sensor structure. This setup analyzes the angles of both the US probe and the robotic arm during scanning and uses a feedback mechanism for the timely correction of the probe’s imaging angle, ensuring good reproducibility of US images. However, such a design can introduce artifacts into the acquired images, compromising image quality. To address this, many researchers have altered the placement of the force sensor. Merouch et al. [56] positioned a force sensor between the flange and the probe for the automatic US scanning of lower-limb arteries, applying a constant force vertical to the patient’s body surface. Mustafa et al. [57] employed a similar design for automated liver US imaging, where the force control algorithm relies on real-time feedback from the force sensor. Fu et al. [89] also installed a force sensor between the flange of the robotic arm and the US probe. This setup acquires contact force data along with the robotic arm’s flange position and velocity to perform an online estimation of environmental parameters. Based on these parameters, the actual contact force in the current state is calculated to ensure the safety of remote US examinations. As shown in Figure 2b, Zheng et al. proposed a low-cost force sensor and force control system for RUS imaging, achieving stable contact through hybrid position–force control [41].

With the increasing demand for human–robot interaction, collaborative medical robots have been developed. These robots typically have force-torque sensors embedded in their joints, which can be used to estimate the Cartesian forces acting on the flange. Many systems directly utilize such robotic arms and apply a constant contact force along the direction of the probe [38,42,53,58,59,60,90]. Lin et al. constructed an RC-RUS using a 7-DOF robotic arm with an embedded force sensor at its flange. They employed Z-direction PD control to maintain a constant contact force of 2.0 N. Comparative experiments with impedance control showed that both control methods could maintain the contact force near the desired value, and differences existed in repeat-scan stability. This work provides reproducible experimental evidence regarding “constant force setting + controller selection” [43].

To prevent potential harm to patients caused by excessive contact force, some researchers have designed specialized end-effector mechanisms to maintain constant contact force, thereby eliminating the need for force sensors and feedback control. Tsumura et al. [61] developed a passive mechanism incorporating a constant-force spring for fetal US scanning, which applies no active force and ensures safety for the pregnant woman and the fetus. Groenhuis et al. [62] designed an acoustically transparent pad for strain elastography reconstruction. No additional force sensor is required as the pad’s elasticity is known priori, and thickness changes can be detected on US images via edge detection. The pressure distribution transmitted through the pad can be calculated and then used to infer pressure within the underlying tissue. Lucas et al. [91,92] designed a soft end-effector consisting of a base and a sensor holder, connected by three soft fluidic actuators arranged in parallel at 120° intervals. This configuration provides two rotations and one translation, achieving passive compliance suitable for US examinations.

To enhance patient safety and mitigate the risk of injury when force-sensor fails, Wang et al. [63] designed a clutch joint (as shown in Figure 2c) that constrains the applied force to a safe range while maintaining the required contact force across different probe poses. As shown in Figure 2d, Sandoval et al. [64] designed a flexible joint coupled to the robot’s flange. This structure is a combination of a linear spring and a multi-joint planar mechanism, providing sufficient compliance to protect patients from excessive contact force. Bao et al. [44] designed a multifunctional end-effector that incorporates a force control mechanism and a force/torque measurement mechanism. This enables the US probe to scan with a constant force while simultaneously measuring the operational forces/torques. The application of a constant force allows for safe US scanning, and the measured forces/torques are used for monitoring and issuing warnings during the procedure. Notably, this multifunctional end-effector operates with independent control, separate from the robotic arm.

The force control methods commonly employed by researchers are predominantly based on admittance controllers, which utilize a predefined relationship between force and position. Carrière et al. [93] used admittance control to ensure compliance in a cooperatively controlled US acquisition system, which regulates the force applied to the tissue and reduces the effort required from the physician. Piwowarczyk et al. [94] proposed an admittance controller to scale the relationship between the force exerted by the physician on the robot and the force applied to the environment. Ferraguti et al. [95] investigated the stability of admittance-controlled robots and their ability to respond to different environmental forces. Dimas et al. [96] analyzed the stability of admittance control by detecting unstable behavior and adjusting the admittance control gains using an adaptive online method to stabilize the robot. Ning et al. [97] proposed a control strategy based on an end-effector admittance controller, achieving simultaneous control of the US probe’s position, pose, and contact force. It is difficult for traditional admittance control to achieve high-precision force control. Therefore, as shown in Figure 2e, Jiang et al. [98] introduced an integral adaptive admittance control strategy, which performs an online estimation of uncertain environmental information and uses the estimated parameters to correct the reference trajectory. While ensuring system stability, it incorporates an integral controller to improve the system’s steady-state response, thereby enabling constant-force scanning in uncertain environments. Xie et al. [99] presented a virtual admittance-based primary–secondary control method, where the controller regulates the contact force between the probe and the tissue, mitigating increases in interactive force to ensure safety. Ning et al. [100] introduced inverse reinforcement learning into the active compliance control of RUS acquisition system, enabling interactive behaviors in uncertain environments that more closely approximate expert strategies. Zhang et al. integrated “dynamic contact force adjustment” with “US image confidence feedback” for robotic breast US scanning, allowing the contact force to be updated online in response to changes in local coupling quality [87].

In summary, the current state of force-sensing research technologies can be primarily classified into three approaches: designing passive mechanisms, attaching external force sensors to the flange of robotic arms, and utilizing collaborative/medical robotic arms with built-in force sensors. Regardless of the method, the constant force applied by the system is often based on empirical data. This force value typically meets the requirement for acquiring US images while ensuring no harm to the patient. However, using a fixed value may lead to varying levels of comfort for different patients or even for different anatomical sites on the same patient. Force control algorithms, predominantly based on admittance control, have achieved favorable results. Nonetheless, it remains necessary to establish a mapping relationship between force control and patient comfort, which plays a crucial role in advancing the acceptance RUS acquisition. In force controller design, three related but distinct requirements should be met. Acoustic coupling defines a minimum contact condition to avoid dropouts and maintain usable images, while safety force thresholds define a conservative upper bound to prevent tissue injury under uncertainty. Patient-perceived comfort is neither identical to coupling nor identical to safety, because discomfort may occur below injury thresholds and varies across subjects and anatomical sites; therefore, comfort needs explicit consideration and reporting.

### 3.2. Scanning Path-Planning and Localization

Scanning path-planning and localization typically involves pre-defining a set of probe positions and poses along planned trajectories to fully cover the target imaging plane or region of interest. The level of autonomy in an RUS acquisition system is closely tied to the effectiveness of its scanning path-planning and localization methods. Accurate and comprehensive planning enables efficient coverage of the imaging plane, thereby precisely acquiring US images of the required structures. This serves as a prerequisite for influencing US image quality.

Some approaches operate without real-time image feedback, relying solely on pre-planned paths within the robot’s workspace. Pahl et al. [66] designed a Cartesian coordinate robot capable of following a pre-designed scanning path without human intervention during operation. However, the system’s insufficient degrees of freedom limited its flexibility, potentially resulting in suboptimal imaging at certain positions. For locating and assessing stenosis to plan treatment for peripheral artery disease, Merouche et al. [56] determined the scanning path by manually delineating it directly along the robotic arm.

Some researchers focus solely on using US images as feedback for path-planning, which is effective but faces several constraints. The lack of objective quality assessment criteria for US images makes it difficult to establish robust decision rules, and such approaches are often only applicable to specific scenarios. For carotid artery diagnosis, Nakadate et al. [65] developed a real-time image processing-based incremental planning approach. Through a pre-designed workflow, it detects carotid artery landmarks, relying solely on US image information for real-time feedback control. Because the image features of the carotid landmarks and the custom-designed scanning strategy are closely tied to the specific characteristics of the carotid artery, it limits the generalizability of this method. Huang et al. [13] divided the carotid artery US scanning process into pre-scanning and scanning to improve its accuracy and efficiency. During the pre-scanning stage, the physician determines the probe’s initial pose based on the RGB-D images. In the scanning stage, the probe’s pose is continuously adjusted by observing real-time US image features to achieve transverse and longitudinal scanning of the carotid artery. Shida et al. [101] modeled the probe’s motion on the body surface as a sequential decision-making problem in the cardiac structure search for transthoracic echocardiography. They employed deep reinforcement learning to generate search behaviors and further utilized a path-generation algorithm to shorten the search path and reduce examination time. This work embodies the concept of end-to-end closed-loop planning, integrating “image–action–path”. Beyond medical US-specific studies, recent mapless navigation research has shown that deep reinforcement learning policies can make motion decisions using only local sensory feedback, without relying on global environment information, while improving generalization across different scenes and even different hardware platforms [102]. Related studies further highlight practical routes toward robust deployment, such as improved TD3-based mapless navigation [103] and Sim-to-Real transfer with minimal sensing [104]. Demonstrations can also be used to accelerate learning and enhance reliability in mapless navigation [105].

Most studies utilize RGB-D cameras to acquire body surface information, which is then registered with the global information of the target tissue obtained from preoperative MRI/CT images. Following this registration, the scanning path is planned on the MRI/CT images. The core of this method involves planning a 2D scanning path in advance and then projecting it onto the 3D surface to complete the scan within a manually selected region of interest. Based on the 3D information obtained through this approach, the surface normal direction of the patient’s body is calculated, ensuring the probe remains vertical to the body surface. Such methods typically combine pre-planning with real-time feedback, allowing for real-time adjustments to the manually defined path. This approach avoids the limitations seen in the methods proposed in Reference [66], which do not account for real-time changes in tissue information. Therefore, both the flexibility and accuracy of AU-RUS can be improved. Jiang et al. [54] proposed a vision-based RUS acquisition system that uses an RGB-D camera to extract manually planned scanning trajectories, followed by estimating the normal direction of the target using the extracted 3D trajectory. This system can monitor the target’s movement and automatically update the scanning trajectory, thereby seamlessly providing a 3D synthesized image of the target anatomy. Wang et al. [106] introduced an image-based probe tracking method to achieve AU-RUS acquisition. By identifying and segmenting preoperative MRI images, an estimation of the probe’s initial pose is obtained, and a transformation from MRI coordinate to US image coordinate is defined to register the MRI images to the real US images. Hennersperger et al. [59] manually planned a scanning path in the patient’s preoperative MRI images by defining start and end points. This path was then transformed into the robot’s workspace through surface registration between the preoperative MRI image and the real-time RGB-D image. Welleweerd et al. [67] proposed using preoperative MRI images to reconstruct the tissue surface or employing CAD files of a model to reconstruct a surface for path-planning, followed by transferring the planned path onto this surface. A similar approach has been applied to various medical scenarios. For instance, in References [68,69] for spinal scanning, and in Reference [70], where the scanning path was planned on a thyroid model and then transferred to the robot’s workspace via registration between the model and real-time RGB-D images. In addition, approaches based on MRI/CT images for pre-planning are also popular. For instance, References [42,59] utilize MRI images for pre-planning, and Reference [38] conducts pre-planning using CT images. Bi et al. [107] addressed the acoustic shadowing problem caused by rib occlusion. They trained a reinforcement learning planner in a virtual environment constructed from CT templates to automatically generate intercostal scanning trajectories that avoid acoustic shadows and cover the target area. This approach aims to enhance coverage efficiency and reproducibility in scenarios with restricted acoustic windows, such as the chest and upper abdomen.

From a clinical feasibility perspective, preoperative CT/MRI-based planning with cross-modality registration can offer strong anatomical priors for target localization and acoustic-window optimization, and is particularly attractive when cross-sectional imaging is already available (e.g., selected preoperative or interventional workflows) [38,42,59]. However, this paradigm typically requires additional steps, such as segmentation, calibration, and reliable registration. Further, its performance can degrade under respiration-induced motion and soft-tissue deformation, which increases workflow complexity and limits scalability in routine diagnostic screening [54,67,106]. Alternately, RGB-D image-based planning combined with online adaptation reduces reliance on preoperative cross-sectional imaging and can better fit near-term clinical workflows due to the lower setup burden and easier integration with real-time perception and compensation [31,32]. Finally, fully autonomous 3D planning and scan completion (including automatic region identification, trajectory generation, and reconstruction in a unified pipeline) is increasingly explored, but in many applications it remains at a research or proof-of-concept stage and still requires stronger robustness, safety-verifiable autonomy, and clinically aligned endpoints before broader deployment [37].

The above path-planning methods all belong to semi-autonomous approaches, such as manually pre-planned paths or manually selected regions of interest, which involve significant human interventions. To enhance the overall system’s autonomy, some researchers have begun to focus on the fully automatic planning of regions of interest or scanning paths. Yang et al. [71] proposed a US system for 3D imaging, which utilizes RGB-D images and a fully convolutional network to achieve the automatic identification of spinal regions of interest and planning of scanning paths. Similarly, Suligoj et al. [108] employed an RGB-D camera to acquire surface point clouds of a human model and performed autonomous path-planning on the curved surface. The obtained images were effectively applied to jugular vein segmentation. Wang et al. [45] aimed to achieve complete and uniform coverage of the breast organ. They utilized an RGB-D camera to acquire point clouds of the breast from multiple angles, registered them together for shape reconstruction, and finally employed an equidistant 3D point cloud search algorithm to complete the path scanning. Tan et al. [37,109,110,111] proposed an end-to-end scanning trajectory generation strategy based on 3D point clouds. Building upon this, they incorporated considerations for trajectory offset strategies and dual-breast synchronous scanning strategies, achieving repeatability in breast scanning. Yang et al. [112] integrated RGB-D and US image information to determine the probe’s path and pose in real-time in spinal scanning. This approach ensures the vertebral structures remain centered in the US image, thereby enhancing the consistency of extended field-of-view imaging and measurements. For abdominal organs such as the kidney, Wu et al. [46] proposed a point-cloud-guided anatomical localization and scanning path-planning strategy, which improved the automation of path generation and its adaptability to individual body surface geometry. Additionally, Sun et al. [47] introduced an AU-RUS system for musculoskeletal US imaging, integrating anatomical region localization, automatic trajectory generation, segmentation, and 3D reconstruction into a unified workflow. The system employs hybrid position–force control to enhance scanning stability and image consistency along the trajectory.

Based on the above research, the current state of scanning path-planning can be summarized into the following four categories: planning within the robot’s workspace without image feedback; planning using only US image feedback; planar-planning based on the registration of preoperative images that are then projected onto the curved surface; and fully autonomous 3D surface path-planning. Currently, path-planning based on preoperative image registration is the mainstream approach, offering sufficient adaptability, and planar-planning avoids the complexities of direct 3D surface-planning. However, the lack of acquisition autonomy remains a non-negligible issue. Therefore, research on fully autonomous scanning path-planning will become the driving force advancing the autonomous progression of RUS acquisition systems.

In practice, scanning path-planning and probe localization for AU-RUS can be separated into four paradigms: (i) preplanned coverage paths (e.g., raster/spiral sweeps) executed with force/impedance regulation; (ii) anatomy-/model-informed planning, where surface/organ priors or protocol templates constrain feasible trajectories; (iii) image-driven online servoing, where real-time US feedback (e.g., target centering or quality surrogates) continuously refines the probe pose; and (iv) learning-based policies that map multi-modal observations to actions to reduce manual intervention. These paradigms differ in robustness, required priors, and failure recovery ability.

Preplanned coverage is simple and reproducible, and thus suitable for large and relatively smooth regions, but it is sensitive to patient motion and anatomical variability. Anatomy-/model-informed planning improves repeatability when reliable registration is available, yet it can degrade under soft-tissue deformation and calibration drift. Image-driven online servoing is effective when the imaging objective can be explicitly defined, but may suffer from noisy feedback and local minima. Recently, learning-based autonomy has shown promising cross-subject generalization, e.g., fully autonomous thyroid scanning and learning-based expert-level carotid ultrasonography, while diffusion-policy learning has also been explored with force-aware constraints for carotid scanning. Nevertheless, safety constraints and clinically meaningful quantitative evaluation remain essential for clinical deployment [34,113,114].

### 3.3. US Treatment Guidance

US-guided therapy enables physicians to focus on the surgical intervention task, allowing for automatic imaging tracking of tools such as needles and catheters to facilitate human–robot collaborative treatment. Compared with diagnostic scanning, interventional guidance requires stricter safety guarantees, lower-latency feedback, and higher failure tolerance, because errors can directly affect tool–tissue interaction and procedural risk. Therefore, interventional systems typically require conservative fallback behaviors and rapid physician override. Langsch et al. [72] proposed an autonomous catheter tracking system for endovascular aneurysm treatment, where the robot’s flange holds a 2D US probe to acquire US images. In preoperative CT scanning, the vascular structures of interest are segmented and then registered to the intraoperative US images. During intervention, the physician inserts the catheter into the abdominal aorta and guides it to the region of interest. The robot employs a tracking algorithm along with force control to follow the catheter, ensuring the catheter tip remains continuously visible in the US image.

For needle guidance and placement tasks, Kojcev et al. [60] proposed a dual-robot system to simultaneously perform US imaging and needle insertion. First, the region of interest is selected from the RGB-D image of the patient’s body surface. During the insertion process, US image-based visual servoing is employed for target-tracking of the needle. Yan et al. [115] proposed integrating visual tracking with motion prediction for the continuous localization of the needle tip in 2D US images. Compared to detection methods solely relying on single-frame appearance features, this approach can still maintain stable tracking even when the needle tip is temporarily invisible or against strong background interference, making it more suitable as a feedback signal for robotic visual servoing.

For carotid artery reconstruction, Faoro et al. [116] proposed a robotic platform for US-guided endovascular surgery. This platform incorporates both preoperative and intraoperative US images, achieving precise 3D vascular volume reconstruction from 2D US image sequences through robotic probe manipulation and AI-based image analysis. Experimental results demonstrate that its reconstruction accuracy reached the submillimeter level. Esteban et al. [117] performed facet joint injections using US- and robot-guided needle insertion for the treatment of chronic back and spinal pain. This work presents the first clinical data on robot-assisted, US-guided facet joint needle insertion surgery, with the results demonstrating the clinical value of the system. Chen et al. [118] proposed a novel convolutional neural network (CNN) framework for the automatic and accurate detection of inserted needles, aiming to enhance the accuracy and success rate of clinical punctures. The method involves segmenting needle motion information to extract two adjacent US image frames, and then extracts the needle’s region of interest from the US images based on the prediction results from the previous frame. This data is then fed into the network, enabling finer and faster continuous needle localization. Grube et al. [119] proposed a deep learning-based needle tracking method for low-resolution volume US and performed quantitative evaluations on a robotically driven acquisition platform, highlighting the potential value of volume US for interventional navigation. Mazdarani et al. [120] integrated confidence maps with visual servoing, enabling the robot to automatically adjust the probe to maintain needle visibility within the imaging plane under conditions of unknown trajectories and complex backgrounds. Their experiments on phantoms/models reported stable tracking accuracy.

US treatment/intervention guidance in autonomous or semi-autonomous RUS can be grouped into: (i) US visual-servoing/confidence-driven tracking, where the robot adjusts the probe to keep the tool or target structure visible; (ii) registration-based guidance, where targets/plans from preoperative CT/MRI are mapped to the US frame; and (iii) integrated scan–localize–guide pipelines, which combine planning, scanning, segmentation/reconstruction, and guidance into a unified workflow. Visual-servoing approaches are attractive for real-time guidance with minimal preoperative imaging, but rely on robust tool visibility and can be challenged by acoustic shadowing. Registration-based methods provide global spatial context but are sensitive to calibration and deformation. Integrated pipelines are promising for reducing operator workload and standardizing workflow, yet require conservative safety design and clinically aligned validation. Recent studies illustrate these directions. For example, confidence-map-based visual servoing has been validated for maintaining longitudinal needle visibility in robotic US-guided PCNL, while an end-to-end autonomous US-guided CVC pipeline has been reported that integrates scan initialization, region/path-planning, vessel reconstruction and supervised needle guidance on high-fidelity phantoms [120,121].

### 3.4. US Image Processing Techniques and Quality Assessment Optimization

Due to the working mechanism of acoustic propagation, US imaging cannot penetrate air or bone. Therefore, the US probe must maintain close contact with the skin, and image acquisition requires selecting an optimal acoustic window while avoiding bone obstruction. Consequently, the relative position between the US probe and the human body is a critical factor affecting image quality. Although advancements in electronic technology, image processing techniques [122], and US transducer design [123] have significantly improved the image quality of current clinical devices, there remains no unified standard for evaluating US image quality.

In this review, “US image quality” for AU-RUS acquisition is interpreted as a clinically usable and protocol-compliant image state, which jointly reflects: (i) physical signal adequacy (e.g., coupling/contact, attenuation/shadowing, signal-to-noise ratio and artifact level), and (ii) task-level diagnostic sufficiency, i.e., whether the target anatomy/plane required by a specific protocol is clearly observable and reproducible. Under this definition, most existing quality assessment methods remain task-specific (e.g., plane-specific scoring for fetal, breast, arthroscopy, or other organ protocols), device- or protocol-dependent (affected by transducer type, frequency, gain, and vendor processing pipelines). Therefore, they are not directly comparable across studies due to inconsistent targets, scoring criteria, and acquisition settings.

Some scholars leverage prior anatomical knowledge of target organs or structures to comparatively assess US image quality, often by referencing the patient’s CT/MRI data [124,125,126]. Another effective approach involves processing the US images directly and evaluating their quality based on the correlation between the processed image content and the target image. Such methods are typically task-oriented, verifying whether the processed images contain the targets necessary for the specific task. For instance, References [73,74,75] proposed automated methods for fetal image quality assessment and fetal biometric measurements in US images. References [76,77] assessed US image quality for breast and knee arthroscopy, respectively. Reference [127] introduced a novel automated conical breast US system for breast cancer detection using a 3D US-MRI fusion method. Experimental analysis indicated that its image quality is comparable to that provided by the Siemens ABVS. Cao et al. [128] proposed and validated an AI-powered automated image quality auditing system for first-trimester screening. This system enables rapid auditing of the imaging quality of key anatomical planes and assists operators in improving acquisition quality. Meanwhile, Liu et al. [129] developed a deep learning-based quality assessment model for the fetal mid-sagittal plane used in nuchal translucency measurement, emphasizing its consistency and usability within multi-source data and clinical workflows.

Furthermore, from the perspective of US physics, more generalized methods for US image quality assessment can be proposed. Some studies estimate the attenuation characteristics of US waves and assess image quality by combining radiofrequency data [130,131,132,133]. Göbl et al. [38] utilized CT images of the target organ to plan scanning paths that optimize the acoustic window using the US attenuation model introduced in Reference [125]. By leveraging the relationship between X-ray attenuation coefficients and US propagation, the US intensity at selected points within the patient’s body can be predicted before performing the actual acquisition task. Furthermore, the optimal probe pose on the tissue surface can be selected to minimize US attenuation. Therefore, based on prior knowledge of the target organ, the planned scanning path can avoid strong reflectors such as ribs, thereby optimizing US image quality during the planning stage. Integrating the principles of US physics, using acoustic window optimization as a criterion is also an effective method. Sutedjo et al. [78] proposed a pose optimization method to address image shadows caused by bone interference during scanning, which effectively improved US image quality.

Some researchers have focused on improving the image quality of RUS acquisition. Part of this work starts from image processing methods, introducing the concept of US confidence maps into US image processing and achieving significant research progress. Karamalis et al. [81] first proposed the concept of US confidence maps, which estimate the confidence level of information described by each pixel in the image. Based on the hypothesis that the probability of US transmission is directly related to the information confidence in the image, and incorporating US-specific constraints, they calculated the probability that a random walk algorithm starting from a given pixel could reach virtual transducer nodes. The random walk algorithm [134] played a crucial role in this process, effectively describing the pixel uncertainty in US images. Building on this foundation, Chatelain et al. [79,80] proposed using the confidence value from the US confidence map as a control signal. They established a link between confidence and the robotic arm within a position-based visual servoing framework, maintaining a constant force between the probe and the patient under an overall redundant control framework to keep the target centered horizontally in the image. Virga et al. [42] employed confidence maps to assess the quality of US images acquired in real-time, enabling the comparison of performances across different control strategies. Welleweerd et al. [67] integrated confidence maps into the visual servoing system of an autonomous breast-scanning robot to adjust the contact between the probe and the patient’s skin, thereby optimizing the quality of the acquired US images. Jiang et al. [3] utilized both US confidence maps and force feedback to estimate the optimal pose of the probe at the contact point, aiming to enhance image quality at a given location. The proposed method seeks to improve US propagation within the tissue by optimizing the US probe’s pose, i.e., aligning the probe’s central axis with the surface normal of the patient at the contact point, and thus addressing the challenges encountered in orthopedic applications.

Additionally, some research focuses on optimizing US image quality during the acquisition process through the real-time updating of the scanning path and adjusting the probe’s pose. Abolmaesumi et al. [82,83,84] proposed several feature extraction algorithms to track the carotid artery in US images in real-time and used a visual servo controller to automatically adjust the probe’s in-plane motion during remote US examinations. Since the US image-based servoing can automatically keep the carotid artery centered in the image, it compensates for unintended patient movement during remote operation. In robotic visual tracking tasks, numerous visual features have been proposed for the use of visual servoing to track targets with complex shapes [135,136,137,138]. TutKunSen et al. [85] proposed a registration-based method that dynamically updates the required probe position in a cooperative system based on the differences between real-time US images and reference US images. Fujibayashi et al. [139] introduced a target image search strategy combining visual servoing and deep learning. This strategy controls the US probe to acquire images at various locations, uses YOLACT++ for anatomical segmentation to extract features, and thereby searches for the optimal kidney US image. To achieve the autonomous visual servoing motion of the US probe, Wang et al. [48] proposed a target-tracking method based on an improved Siamese network. This provides real-time dynamic feedback control of the probe by analyzing the differences between a template image and real-time US images, aiming to acquire high-quality images. Furthermore, to prevent image loss during scanning, they utilized the average intensity of the US images to characterize the coupling relationship between the probe and the scanned tissue. Tang et al. [140] integrated pose recognition with image-based servoing control in their autonomous cardiac US acquisition system. By assessing low-quality regions, the system triggers acoustic window/pose corrections, thereby enhancing the success rate of acquiring high-quality cardiac images. Meanwhile, Lin et al. [43] compared the effects of different control strategies on repeatability and consistency within an autonomous US acquisition system. Their work further validates the critical role of visual servoing and real-time correction in consistently obtaining analyzable images.

In summary, the current research on US image quality assessment and optimization can be briefly categorized into five types: (1) quality assessment by incorporating anatomical prior knowledge; (2) quality assessment by integrating US attenuation models; (3) quality assessment and optimization using US confidence maps; (4) image quality improvement through acoustic window optimization; and (5) real-time path adjustment via visual servoing to enhance image quality. Due to the current lack of a unified, objective evaluation system and relevant metrics for US images, each research method has its own distinct characteristics, making it difficult to determine absolute superiority or inferiority. Selecting an effective assessment approach based on specific technical solutions and application scenarios can meet certain research needs. However, it remains evident that the absence of a unified, objective US quality evaluation system significantly hinders the advancement of US image quality improvement. More importantly, the absence of standardized, task-agnostic and cross-device comparable quality metrics becomes a fundamental bottleneck for autonomous decision-making, because it limits the design of robust quality-aware feedback rules (e.g., re-positioning, termination, and failure recovery) and hinders fair benchmarking and multi-site clinical validation.

## 4. Limitations and Challenges of Current Research

Leveraging robotic assistance, US acquisition is now capable of replacing physicians in certain medical scenarios. In terms of effectiveness, current RUS acquisition systems can achieve image quality with higher standardization and consistency than that achieved by physicians, and they are gradually being applied in clinical diagnosis and anatomical structural biometry. However, as current AU-RUS systems remain in an ongoing exploration and research phase, technical limitations are also obvious in these systems.

From the deployment viewpoint, we prioritize the challenges into near-term critical bottlenecks and longer-term goals. Near-term bottlenecks include safety-verifiable autonomy with reliable failure detection and recovery, standardized and clinically meaningful image quality metrics for objective validation, and motion compensation under non-rigid deformation. Longer-term goals include broad cross-organ generalization and higher-level autonomy toward diagnostic decision support.

### 4.1. The Balance Between the Need for Enhanced Robot Autonomy and Human Safety

As evidenced by numerous studies discussed above, AU-RUS acquisition technology is capable of replacing traditional physician-held manual operation. Currently, most research focuses on SA-RUS systems, which require collaboration between humans and robots during the acquisition process, with the entire procedure still necessitating physician adjustment and control [67,141,142,143]. In some systems, the initial probe position is also determined by the physician. Even for AU-RUS systems, the initial application of the acoustic coupling gel at the beginning of the examination is performed manually. Compared to semi-autonomous systems, autonomous acquisition systems eliminate the need for human participation, thereby significantly reducing the physician’s workload. Hence, enhancing robot autonomy is essential. With the increasing shortage of medical resources and the ongoing global pandemic, the demand for US examinations continues to rise. However, US examination heavily relies on the physician’s expertise and experience, and the number of skilled sonographers cannot meet clinical needs. It is still difficult for families and individuals with special requirements in remote areas to receive timely, professional US examinations. Therefore, there is an urgent need for fully autonomous RUS examination systems [144]. However, as the level of robot autonomy increases, the demands on the robot extend beyond mere operation to include more sophisticated perception and decision-making. To achieve this, it is necessary to enhance the robot’s perception and decision-making capabilities through algorithmic innovations, enabling it to respond to various sensor data and make correct decisions, thereby better supporting physicians in US acquisition.

The enhancement of robotic autonomy inevitably raises concerns about safety. The people-centered philosophy makes safety the primary issue determining whether AU-RUS systems can be widely adopted. Increased autonomy implies greater medical risks due to a higher possibility of failures [145], requiring the system to possess adaptive adjustment mechanisms. It must be capable of responding to and quickly adapting to unexpected situations, maintaining system stability and ensuring that the force applied by the probe remains safe in the presence of parameter uncertainties and external disturbances [146]. Although many systems incorporate force sensors or passive mechanisms to guarantee safe force application, the inherent possibility of sensor failure inevitably introduces risks. Enhancing real-time fault detection for sensors can improve safety to some extent, but the consequent burden of extensive data processing also becomes a challenge. Zheng et al. proposed a low-cost force sensor and a hybrid position–force control strategy to reduce the system’s cost barrier while maintaining contact safety [41]. Wu et al. developed an adjustable constant-force end-effector [147]. By combining active long-stroke compensation with a passive constant-force compliance buffer, the system enhances contact force stability and tolerance to variations in body surface geometry.

The autonomy–safety trade-off is largely driven by uncertainty in (1) perception (poor acoustic windows, speckle noise, and out-of-distribution anatomy), (2) contact mechanics (patient-dependent stiffness/friction and non-rigid deformation), and (3) system drift/latency (calibration drift and delayed feedback). These uncertainties can lead to unsafe contact forces or loss of acoustic coupling. Therefore, beyond improving nominal performance, a key bottleneck is robust failure detection and recovery, e.g., recognizing target loss, slip, unexpected patient motion, or sensor faults, and triggering conservative fallback behaviors. Recent constant-force end-effector designs with hybrid active–passive mechanisms also highlight the importance of hardware-control co-design for maintaining safe contact under changing surfaces [148].

Furthermore, the inherent safety risks associated with the system’s rigid hardware cannot be overlooked. Advances in soft robotics technology offer an effective approach to enhance human–robot interaction safety by integrating flexible materials with US at the end-effector. By meeting the necessary stiffness requirements, the inherent characteristics of soft fluidic actuators can establish a safe and adaptable interaction between the US probe and the patient [92].

In addition to focusing on tangible physical safety, we must also consider intangible psychological safety, e.g., whether patients can accept a robotic physician. Therefore, human safety should also take into account patient comfort and acceptability. Current research has not incorporated patient feedback as a study metric. To further promote the clinical and community acceptance of AU-RUS acquisition, patient feedback represents a crucial factor for consideration.

### 4.2. Generalization of Application Scenarios

The insufficient compatibility of AU-RUS systems across diverse scenario requirements may be a significant factor limiting their potential to lower the threshold of healthcare access and promote community-wide dissemination. On one hand, different application scenarios share certain common requirements. For instance, the fundamental requirement is the visibility and structural clarity of the imaged target. A universal technique involves image processing to extract structural features. For organs with simple anatomy and favorable locations, this may suffice to meet task demands and yield satisfactory US images. On the other hand, as outlined in the preceding literature review, autonomous robotic systems have been applied for US acquisition in a wide range of medical scenarios. Target anatomical structures include the lung [32], thyroid [34], carotid artery [13], breast [149], liver [150], cervix [151], fetus [152], lower-limb arteries [153], and kidney [139]. Each application possesses distinct examination characteristics and image acquisition requirements. AU-RUS systems developed based on general scanning principles cannot be specifically tailored to all unique medical contexts. This is also a major reason why such systems struggle to achieve full autonomy. Therefore, enhancing the system’s perceptual and decision-making capabilities, enabling it to autonomously adapt to different medical scenarios, is essential for effectively promoting the broader application of RUS systems.

Furthermore, advancements in hardware design present a significant challenge. For different organs, the types of auxiliary sensors required for US acquisition may vary, and the corresponding US transducers may also differ. Current research tends to be highly specific, with individual systems typically applicable only to particular organs and lacking generalization capabilities. Although different organs have distinct requirements for sensor types and imaging parameters, the development of unified, generalized equipment capable of adapting to diverse scenarios holds substantial importance for clinical adoption and widespread implementation.

### 4.3. Human Motion and Respiratory Compensation

Most existing robotic systems for autonomous US acquisition require patients to hold their breath or remain still during scanning [37]. However, this requirement can be uncomfortable and inconvenient for patients. Although some current systems incorporate compensation functions for small-scale patient movements, they may fail to operate properly if unexpected or substantial movement occurs during the examination.

Real-time observation and tracking compensation for patient motion and tissue deformation are beneficial for enhancing system stability and improving patient comfort and experience during medical procedures, particularly in intraoperative imaging applications. Many systems employ visual servoing methods for real-time control of the US probe, enabling real-time tracking based on visual detection or image information [154] to compensate for tissue motion in medical scenarios. Various features in US images are utilized for tissue tracking, including speckle information [155,156,157], image moments [158], and intensity [159,160], further improving imaging performance during tissue movement.

Motion compensation remains difficult because US acquisition couples probe motion, tissue deformation, and imaging quality in a highly nonlinear manner. Respiratory motion is quasi-periodic but patient-dependent, while soft tissue exhibits non-rigid deformation that cannot be removed by rigid tracking alone. A further bottleneck is real-time coordination between motion prediction, force/admittance control, and volume reconstruction; otherwise, stable contact may conflict with accurate spatial compounding. Recent work has explored respiratory-motion-robust RUS using vision–haptic fusion control with predictive compensation, and breathing-compensated 3D reconstruction using implicit neural representations for RUS screening [161,162].

### 4.4. Real-Time Cognition and Quality Assessment of US Images

The rapid development of AI presents new opportunities and challenges for AU-RUS systems. Cutting-edge algorithms from the fields of computer vision and image analysis are increasingly being applied to US acquisition, fostering expectations regarding the intelligent capabilities of US robots. These robots are gradually evolving from precise tools into intelligent agents. In particular, recent studies increasingly treat AU-RUS acquisition as a closed-loop “perception–control” problem, where real-time image understanding directly supports probe motion-planning and adjustment [4]. Data-driven intelligent algorithms such as deep learning are progressively being integrated into image analysis, yielding state-of-the-art results in tasks such as the classification, detection, and segmentation of various anatomical structures [163]. These capabilities can be extended to online view/anatomy recognition and image-quality estimation, providing direct feedback for real-time probe guidance [4]. Image-based probe guidance is increasingly being employed in machine learning methods, with some algorithms already being applied in simulated US acquisition for fetal [164] and cardiac imaging [140]. In addition, multi-modal learning (e.g., combining US video with probe motion and sonographer gaze) has been explored to better model expert scanning behavior and provide guidance signals for navigation [165]. Ning et al. [166] proposed an AU-RUS system that utilizes reinforcement learning to achieve the adaptive constant-force tracking of soft moving targets with the US probe. Building on this, they introduced a force-position control method based on an admittance controller, achieving autonomous control of the probe. Li et al. [167] proposed a deep reinforcement learning solution that controls the US probe towards the desired imaging plane based on real-time US image feedback. Related work has further explored reinforcement learning for standard-plane localization in practical scanning tasks. Li et al. proposed an RL-based RUS system for the automated localization of standard liver planes using a DQN-LSTM agent and reported promising image and anatomy localization metrics [168]. In addition, Si et al. proposed a deep multimodal imitation learning framework that fuses RGB and US images, force signals, and robot proprioception to predict desired probe motion and contact force, and executes the learned skill using compliant control and trajectory optimization [169]. In future research, robotic systems are expected to integrate image acquisition, cognition, decision-making, and guided probe movement, ultimately better assisting US physicians in performing US acquisition.

Real-time cognition requires evaluation standards for quality assessments to effectively function and for interpretation of the results. However, at present, there is no unified evaluation standard, let alone quantitative metrics. Recent clinically oriented studies also emphasize protocol-driven evaluation and reproducibility reporting, which further motivates standardized quantitative metrics for quality assessment [4,114]. Furthermore, many studies only qualitatively validate the effectiveness of the proposed methods or systems, lacking the ability to objectively and quantitatively evaluate their performance. Therefore, establishing a quantitative and standardized quality assessment system represents a crucial future research direction and challenge, enabling the validation of methods and systems based on clinical application outcomes.

Encouragingly, recent studies have started to move towards more quantitative and benchmark-oriented quality assessments. For instance, fully autonomous thyroid scanning systems reported multiple quantitative indicators related to contact condition and target centering/pose, providing a reproducible way to evaluate autonomous scanning quality. In addition, benchmark efforts such as Ultrasound-QBench have been proposed to assess model capabilities regarding US image quality (classification/scoring/comparative assessment), which may inspire more standardized evaluation pipelines. Moreover, domain-specific quality assessment models for robot screening have also been reported recently, indicating a trend toward quantitative, task-aligned metrics for RUS acquisition [34,170,171].

## 5. Perspective of Techniques for AU-RUS Acquisitions

The development trends in RUS focus on enhancing autonomy in image acquisition, diagnosis, and treatment guidance. For instance, autonomous planning technologies are replacing the manually planned components required in semi-autonomous systems. Further, the ability to compensate for target motion and deformation should be improved to prevent the loss of target visibility in US images. The integration of US robots into clinical workflows and the promotion of community services are also subjects of ongoing research. In this context, the interaction between the robot and the patient, along with safety aspects, must be ensured. Additionally, AI technologies can be leveraged for image processing and analysis, enabling real-time interpretation and diagnosis.

### 5.1. Enhancement of Cognitive Judgment and Autonomy Through AI Technology

A current challenge in RUS acquisition systems lies in the cognition and understanding of US images. To achieve more significant acquisition outcomes, it is essential to fully integrate image processing techniques and advance research on technologies for image cognition and judgment that are specifically tailored to US images.

From the perspective of this paper, AI has two primary application domains for improving the acquisition effectiveness of future RUS systems: image understanding and robotic navigation planning. Regarding image understanding, CNNs have demonstrated exceptional performance in medical image analysis [172,173,174,175,176] and have been successfully applied to US images [177,178,179,180]. Jiang et al. [114] introduced UltraBot, a learning-based autonomous carotid US robot. The system simultaneously acquires anatomical awareness and scanning skills based on a unified imitation learning framework trained on large-scale expert demonstrations. In clinically oriented validation, it demonstrated a high success rate and achieved expert-level agreement. Intelligent image understanding can be utilized for image quality assessment [74] or for medical measurement and diagnosis [181,182,183]. Concerning robot navigation and planning, deep reinforcement learning [184,185,186,187] has achieved breakthroughs in robot learning in, for example, perceptive path-planning and navigation [188], and real-time obstacle avoidance in complex dynamic environments [189,190]. For example, cross-platform mapless DRL navigation without global information has been reported to transfer across different scenes, which provides a useful reference for designing more robust learning-based probe navigation under partial observability [102]. These methods may play a key role in solving the task of autonomous US probe placement and could be applied in US-guided robot navigation and positioning, e.g., in the autonomous localization of standard fetal facial planes [191].

Furthermore, reinforcement learning, with its characteristic of learning policies through interaction with the environment to maximize reward, has garnered significant attention from researchers. It establishes a relationship between the environment and the system. By learning the relationship between contact force and output force, and defining the desired output force as the workspace, reinforcement learning can enable action generation under visually constrained conditions for soft, uncertain environments. Compared to traditional visual planning and force control methods, this allows for simultaneous position, pose, and force control without requiring prior knowledge, while also avoiding issues like occlusion and parameter tuning [97]. This mechanism of reinforcement learning highlights its potential for application in training novice physicians. An end-to-end trained model can filter out ambiguous regions and guide novices in selecting optimal acoustic window positions to acquire clear cardiac images.

Additionally, reinforcement learning can be used to imitate the operational patterns of experienced sonographers, enabling robots to progressively learn expert-level skills. By combining imitation learning with reinforcement learning, robots can master more complex techniques, such as fine-tuning probe pressure and optimizing imaging angles, thereby enhancing the stability and precision of examinations. However, given the variations in patient physique and lesion locations, achieving generalization capabilities in such systems would require a multiplicative increase in the data volume and computational load for reinforcement learning, and the scanning strategy would also need to adapt as patients change. In a recent review, Bi et al. [192] systematically summarized the key bottlenecks of machine learning methods in RUS: there are high costs associated with data acquisition and annotation, insufficient generalization across devices and populations, and a lack of safety constraints and interpretability. They further pointed out that future efforts need to more tightly integrate quality assessment, strategy learning, and safety control into a unified closed-loop framework.

Although some AI technologies have demonstrated experimental success during application, their current limited interpretability and reliability make clinical adoption challenging. Nevertheless, these technologies can be integrated with reliable control methods to play a role in image interpretation, thereby enhancing the effectiveness of US imaging.

### 5.2. Virtual Reality and Augmented Reality Technologies

Regarding virtual reality (VR), US data can be displayed on a graphical user interface for navigation [193]. Virtual scenes, enhanced by incorporating 3D models of the robot, enable robot-controlled US probe guidance for therapeutic purposes [194], as well as the simulation and verification of robotic equipment [44].

Augmented reality (AR), it has attracted considerable attention in recent years due to its outstanding information fusion abilities, which enables the effective augmentation of reality. In medical settings, AR can provide physicians with critical real-time information and enhance spatial awareness, thereby improving procedural accuracy and reducing the risk of errors [195]. In US acquisitions, AR can overlay 2D US images [196], 3D US images, and reconstructed US phantoms onto real-world scenes, allowing physicians to acquire US images while simultaneously observing the patient; thus, it has strong potential to improve ergonomics. In particular, in RUS scenarios, AR-based overlays can enhance physicians’ perception of critical anatomical structures, improve 3D understanding, and simplify hand–eye coordination, thereby improving the user experience [197]. Beyond clinical assistance, AR can also be used for skills training and simulation-based practice. By superimposing virtual information onto the real world, AR can create flexible and controllable training scenarios and provide sensory feedback and interaction experiences that more closely resemble real operations, thereby supporting procedural training such as US-guided needle puncture. For example, in training for US-guided renal biopsy, both VR and AR platforms can help alleviate practical challenges, including the high of difficulty in the training, low efficiency, and the inability to repeatedly practice on human subjects [198]. With advances in US probes and nonlinear image registration, VR/AR presents new opportunities for RUS in visualization, sensor integration, and user interaction.

### 5.3. Soft Robotics Technology Ensuring Compliance and Safety

In RUS acquisition systems, a critical prerequisite is ensuring the safety of human force interaction. Maintaining a balance between the force needed for effective image acquisition and ensuring human safety is an important research direction. Soft robotics technology represents a key avenue for safe human–robot interaction. The compliant materials of soft robots inherently offer a degree of safety, while research into rigidity–softness coupling and variable stiffness enables them to possess sufficient load-bearing capacity to meet the demands of US acquisition.

The design and control techniques of end-effector grippers incorporating soft materials fully leverage their advantages. Compared to rigid devices, they offer higher compliance; for instance, the operational behavior of parallel mechanisms more closely resembles that of the human wrist [92]. Several researchers have developed various flexible mechanisms [199] to provide passive compliance in driving robotic joints.

In recent years, soft robotics technology, by utilizing deformable materials and structures, has opened up new design paradigms for robotic systems. In medical scenarios such as surgery, soft robots often exhibit highly flexible operational capabilities but lack sufficient load-bearing capacity. To find a balance between mobility and load-bearing capability, some researchers have conducted studies on soft robotic systems with variable stiffness capabilities, and demonstrated some progress. These systems have been applied in delicate surgical procedures, including pericardial puncture [200], providing a solid technical foundation for further integration with US imaging technology.

### 5.4. Integrated All-in-One Acquisition–Processing–Diagnosis Solution

Most current systems focus solely on the task of US acquisition. The objective of the research should be to reduce human labor. Therefore, if the entire acquisition–processing–diagnosis workflow can be seamlessly integrated, the system will achieve a high level of intelligence, allowing the US physician to transition into the role of a supervisor and evaluator.

The diagnostic component of this workflow is inseparable from AI assistance. Deep learning and reinforcement learning can learn from vast datasets of image cases and, through continuous training, realize diagnostic functions. These include liver disease classification [201], COVID-19 classification [202], vagus nerve detection [203], and breast tumor classification [204], among others. The realization of this technology would significantly lower the barriers to using US, effectively address the issue of the uneven distribution of medical resources, promote the deployment of US examinations at the community level, and achieve high efficiency in resource utilization within smaller regions.

### 5.5. From Laboratory Systems to Clinical Translation

While AU-RUS systems have shown rapid technical progress, clinical translation requires a pathway beyond algorithmic performance. First, standardization and verification should be established for calibration, safety force limits, image quality targets, and repeatability across operators and sites. In addition, workflow scalability is a practical determinant of clinical adoption: approaches relying on preoperative CT/MRI and cross-modality registration may be best suited to settings where such imaging is already routine, whereas RGB-D surface-based planning with online adaptation is generally more compatible with near-term deployment due to the reduced setup and coordination burden [13,31,32]. Second, human–computer interaction must be designed for the clinical workflow, including the intuitive visualization of system confidence, shared-control modes, and rapid physician override, following usability engineering principles. Third, deployment requires training and competency building for sonographers/physicians to integrate semi-autonomous workflows safely. Finally, regulatory and compliance considerations (risk management, usability engineering evidence, and appropriate submission pathways) are critical barriers. In the European Union and the United States, Medical Device Regulation (MDR) and the Food and Drug Administration (FDA) emphasize safety/performance validation and human-factors engineering, which should be incorporated early in the system design process.

## 6. Conclusions

Leveraging the complementary strengths of robotics and US imaging, an increasing number of RUS acquisition systems have been developed, achieving the automation of RUS scanning across a wide range of medical applications. The progress in AU-RUS acquisition has demonstrated the potential for robots to autonomously obtain reproducible and diagnostically usable imaging results without the need for specialized operators.

This paper first analyzes the advantages and limitations of current RUS imaging technologies. Following a comparison of the characteristics of teleoperated and autonomous systems, it highlights the research necessity for AU-RUS systems and introduces several representative systems. Subsequently, it provides an overview of the current research landscape from four key technical perspectives: force-sensing and control, scanning path-planning and localization, US-guided therapy, and US image processing techniques for quality assessment and optimization. This review presents the latest systems and technologies for AU-RUS acquisition, applied in various clinical contexts. Based on the state-of-the-art offered by existing robotic systems, the paper discusses the shortcomings and challenges in the current research. Finally, it presents a future outlook for AU-RUS acquisition from multiple perspectives.

Current efforts in AU-RUS acquisition are crucial for promoting the community-based deployment of US healthcare. However, future progress in AU-RUS will depend not only on reliable mechanical execution, but also on robust perception, quality-aware decision-making, and standardized, clinically meaningful US image quality assessments to support safety-verifiable autonomy and multi-site clinical validation. With the advances in other related fields, such as soft robotics and AI-powered US image analysis, AU-RUS is expected to play an increasingly prominent role in a broad spectrum of clinical applications.

## Figures and Tables

**Table 2:** Summary of RUS acquisition techniques.

Technique	Implementation	Key Features	References
Force sensitivity and control	Probe force sensors maintaining 1~8 N range	Inherent shadowing at image edges	[39,40]
	Flange 6-axis force/torque sensor	Precise constant flange force	[56,57]
	Built-in force/torque sensors	Force-feedback-based compliant control	[38,53,58,59,60]
	Built-in sensors for patient-specific optimal force	Contact force optimization via US confidence maps	[42]
	Constant-force spring (passive mechanism)	Mechanically guaranteed force safety	[61]
	Force computed via acoustically transparent pad	Mechanically guaranteed force safety	[62]
	Clutch joint limiting force range	Mechanically guaranteed force safety	[63]
	Linear spring and flexible joint for safe force	Mechanically guaranteed force safety	[64]
Scanning path-planning and localization	Real-time US-based path-planning	US image-only control	[65]
	Manual path-planning in robot workspace	Fixed pre-planned paths (non-adjustable)	[66]
	RGB-D-based adjustment of planned paths	Real-time RGB-D tracking of planned paths	[54]
	Preoperative path-planning	CT, MRI, CAD models with image registration	[42,51,59,67,68,69,70]
	Automatic path-planning from surface point clouds	Selected ROI surface point clouds	[45,71]
US treatment guidance	Catheter tracking via preoperative anatomy and US	3D vessel registration and interventional 3D US	[72]
	US imaging + visual/force-guided needle insertion	Dual-arm coordination + pre-/intra-op image registration + depth vision navigation	[60]
	US-guided spinal needle insertion	US-based servoing for spinal needle insertion	[47]
Image processing techniques, quality assessment and optimization	Image quality assessment using anatomical priors	Biological parameter comparison	[73,74,75,76,77]
	RF data and US attenuation-based quality assessment	US attenuation characteristics	[73,74,75,76]
	Acoustic window optimization to reduce shadowing	Acoustic window optimization	[38,78]
	Real-time probe adjustment via US confidence maps	US confidence map	[3,79,80,81]
	Visual servoing for path adjustment	Preoperative image registration	[82,83,84,85]

## Data Availability

No new data were created or analyzed in this study.
